# Measurement properties of the UK-English version of the Pediatric Quality of Life Inventory™ 4.0 (PedsQL™) generic core scales

**DOI:** 10.1186/1477-7525-3-22

**Published:** 2005-04-01

**Authors:** Penney Upton, Christine Eiser, Ivy Cheung, Hayley A Hutchings, Meriel Jenney, Alison Maddocks, Ian T Russell, John G Williams

**Affiliations:** 1 Department of Psychology, University of Sheffield, UK; 2 Swansea Clinical School, University of Wales Swansea, UK; 3 Department of Child Health, Cardiff and Vale NHS Trust, UK; 4 Department of Community Child Health, Swansea NHS Trust, UK; 5 Institute for Medical & Social Care Research, University of Wales Bangor, UK

## Abstract

**Background:**

Health related quality of life (HRQL) has been recognised as an important paediatric outcome measurement. One of the more promising measures to emerge in recent years is the Pediatric Quality Of Life Inventory (PedsQL™), developed in the US. Advantages of the PedsQL™ include brevity, availability of age appropriate versions and parallel forms for child and parent. This study developed a UK-English version of PedsQL™ generic module and assessed its performance in a group of UK children and their parents.

**Methods:**

PedsQL™ was translated to UK-English. The psychometric properties of the UK version were then tested following administration to 1399 children and 970 of their parents. The sample included healthy children, children diagnosed with asthma, diabetes or inflammatory bowel disease and children in remission from cancer.

**Results:**

Psychometric properties were similar to those reported for the original PedsQL™. Internal reliability exceeded 0.70 for all proxy and self-report sub-scales. Discriminant validity was established for proxy and self-report with higher HRQL being reported for healthy children than those with health problems. Sex differences were noted on the emotional functioning subscale, with females reporting lower HRQL than males. Proxy and self-report correlation was higher for children with health problems than for healthy children.

**Conclusion:**

The UK-English version of PedsQL™ performed as well as the original PedsQL™ and is recommended for assessment of paediatric HRQL in the UK.

## Background

Advances in medical research have changed the emphasis in healthcare from diagnosis and management of infectious disease to prevention and control of chronic conditions. While there have been major advances in treatment of previously life threatening conditions (e.g. cancer, cystic fibrosis), treatments can be aggressive and associated with both acute, and long-term morbidity. Recognition of this has led to a shift from measuring efficacy of treatment purely in terms of survival, to one that also takes into account the quality of the resulting life. As a consequence, a number of measures of health related quality of life (HRQL) have been published. Many are based on the definition of HRQL described by the World Health Organisation (WHO), and include separate measurement of physical, emotional and social functioning. A recent systematic review [1] concluded that one of the more promising measures for children was the PedsQL™ [2]. Developed in the US, the advantages of PedsQL™ include brevity, availability of age appropriate versions and parallel forms for child and parent.

PedsQL™ integrates generic core and disease specific modules into one measurement system. PedsQL™ 1.0 [2] was described as a generic instrument. This was developed from work with children with cancer but designed for use as a non-categorical instrument. Subsequent publications have reported several refinements to the generic measure. PedsQL™ 2.0 and 3.0 included additional constructs and items, a more sensitive rating scale and a broader age range. PedsQL™ 4.0 included further core dimensions to match those described by WHO. Recent reports confirm the reliability and validity of this generic measure [3,4]. The success of PedsQL™ can be seen in its wide use in research and translation into many European and other international languages. In this paper we report the performance of the UK-English version of PedsQL™ 4.0 generic core module in a sample of healthy children and children with chronic health conditions.

## Methods

### Measures

PedsQL™ includes parallel child self-reports (age range 5–18 years) and parent/carer proxy-reports (age range 2–18 years). Items on self and proxy-report are virtually identical, differing only in developmentally appropriate language and first or third person tense. Instructions ask how much of a problem each item has been during the past month and responses are made on a five-point scale ranging from 0 (never a problem) to 4 (almost always a problem). The generic module comprises twenty-three items that contribute to four subscales: Physical Functioning, Emotional Functioning, Social Functioning and School Functioning. It has also been shown that Physical Functioning can be viewed as a distinctive scale, while the remaining subscales can be more parsimoniously viewed as a single Psychosocial Health Summary Scale [3]. A Total Scale score can also be calculated. We employed self-report forms for ages 8–18 years and parallel proxy forms.

Translation followed recommended guidelines [5,6]. Preliminary changes to the original questionnaires were made by three experienced psychologists and reviewed by Dr Varni, who recommended further modifications. The revised questionnaires were administered to 13 parents and 22 children and cognitive interview techniques [6] were used to obtain feedback about the interpretation and understanding of items and response ratings. Further changes were made to the questionnaires in response to feedback from parents and children. Dr Varni reviewed the revised measure and authorised all changes.

### Procedure

Healthy children and their parents were recruited through 23 schools in South Wales. Written information was sent to parents who completed questionnaires at home, returning them to school by a specified date, along with signed consent for their child's participation. Children were given verbal and written information before completing questionnaires in class, under the supervision of a researcher.

Children with either asthma, diabetes, inflammatory bowel disease (IBD) or in long-term remission from cancer were identified through patient information systems. Families were informed about the study by post and arrangements made for those who consented, to complete questionnaires either in clinic or at home under the supervision of a researcher.

In addition to PedsQL™ all families completed a brief questionnaire concerning demographic information and child health. Based on this, children with any chronic health problems were excluded from the schools sample, ensuring this group was healthy.

### Analysis

Items on PedsQL™ were reverse scored and linearly transformed to a 0–100 scale, with higher scores indicating better HRQL.[3] Scale scores were created by dividing the sum of responses by the number of items answered (to account for missing data). Internal reliability was assessed using Cronbach's Alpha [7] and range of measurement was determined based on the percentage of scores at extremes of the scaling range [8]. Discriminant validity was evaluated through a comparison of healthy children and those with chronic health conditions. A multivariate analysis of variance (MANOVA) was undertaken in order to determine differences in sub-scale ratings depending on child age, sex or health status. Pillai's Trace was calculated as this is robust to departures from normality. The source of significant variance was then located by Analysis of Variance (ANOVA). Finally the relationship between self and proxy-report was assessed by correlation.

### Ethics

The Welsh Multi-centre Research Ethics Committee gave ethical approval to this work.

## Results

### Sample

From 2002 families approached, a total of 1399 were recruited to the study (response rate = 69.88%), with 1034 families being recruited from schools and 365 from clinics. The remaining 603 families either failed to return questionnaires to schools by the cut-off date (N = 349) or did not reply to letters from clinics inviting participation in the study (N = 254). The sample was homogenous in ethnic background with 90% having been born in the UK and describing themselves as British. All participants either had English as their first language (96%), or were bilingual in English and Welsh (4%). 34% of mothers had left school at 16 (15% with no formal qualifications, 19% with some GCSEs) 39% had completed further education and 22% had qualifications from higher education. Only 5% of the sample did not provide this information. All children were aged from 8–18 years (mean age for self-report = 12.58, sd = 2.6; mean age for proxy-report = 11.86, sd = 2.3). Self-report forms were completed by 684 males and 715 females and proxy-reports were completed by 459 parents of males and 504 parents of females. A complete breakdown of the sample is given in table 1.

**Table 1:** Summary of recruitment and questionnaire completion by child health

	Healthy	Diabetes	Asthma	IBD	Cancer survivor	Total
Number of self- reports completed	1034	124	99	76	66	1399
Number of proxy-reports completed	665	103	74	67	61	970

The difference in self and proxy-report completion shown in table 1, resulted from parents (N = 429) who gave consent for their child to complete PedsQL™, but did not complete a questionnaire themselves. The majority of proxy-reports were completed by mothers (84%), the remaining forms being completed by fathers (14%) or other carers such as stepparents and grandparents (2%).

### Internal reliability

All self and proxy-report sub-scales exceeded the minimum standard of 0.70, whilst the total score exceeded 0.90.

### Range of measurement

The full range of 0–100 was used for all four proxy-report subscales and the majority (3/4) of self-report subscales. A range of 10–100 was used for Emotional Functioning on the self-report, with nobody scoring at the lowest end of this sub-scale. Table 2 presents scale means and percentage of scores at the floor and ceiling for the original PedsQL™ [3] and the UK-English version. No floor effects were seen on the UK-English self or proxy-report for healthy children or those with known health conditions as no scale had more than 0.3% scoring at the minimum. However, ceiling effects existed for the healthy sample and ranged from minimal (e.g. 3.2% and 3.6% for self and proxy-report, respectively for Total Score) to moderate (e.g. 41.4% and 37.4% for self and proxy-report, respectively for Social Functioning). Ceiling effects also existed for those with known health conditions; as for the healthy sample the largest effect was for Social Functioning (35.3% and 26.3% for self and proxy-report). Healthy children and their parents reported more ceiling effects than those with health problems. As table 2 demonstrates, patterns of ceiling and floor effects are similar, to those reported for the original PedsQL™ [3], although ceiling effects are smaller in the UK population. Scale means are also similar on both versions of the measure.

**Table 2:** Comparison of scale statistics for UK-English and original PedsQL™ [3] 4.0 self and proxy-report

Scale	**Scale statistics**					
	Mean (SD) Total UK Sample	Mean (SD) Total US Sample	Percentage floor chronic health condition/healthy (UK Sample)	Percentage floor chronic health condition/healthy (US Sample)	Percentage ceiling chronic health condition/healthy (UK Sample)	Percentage ceiling chronic health condition/healthy (US Sample)
**Self-report**						
Total score	82.25 (13.09)	79.62 (15.26)	0.0/0.0	0.0/0.0	1.4/3.2	1.9/7.2
Physical health	86.08 (14.06)	80.19 (19.30)	0.3/0.0	0.0/0.0	12.1/20.5	13.1/25.8
Psychosocial health	80.50 (14.06)	79.37 (15.70)	0.0/0.0	0.0/0.0	2.5/4.1	5.2/12.0
Emotional functioning	76.99 (18.43)	78.10 (20.66)	0.0/0.0	0.3/0.8	4.9/15.6	22.4/29.8
Social functioning	86.85 (16.86)	84.09 (18.50)	0.3/0.2	0.0/0.0	35.3/41.4	33.2/47.1
School functioning	77.29 (16.92)	75.87 (19.71)	0.0/0.1	0.3/0.5	8.2/11.1	13.0/23.1
**Proxy-report**						
Total score	81.12 (13.85)	80.87 (16.73)	0.0/0.0	0.2/0.0	0.7/3.6	4.1/10.3
Physical health	84.99 (16.08)	81.38 (23.18)	0.0/0.1	2.3/0.0	7.4/26.7	18.5/39.6
Psychosocial health	79.00 (14.70)	80.53 (16.52)	0.0/0.0	0.2/0.0	1.7/4.6	5.6/13.8
Emotional functioning	74.67 (17.67)	77.95 (20.67)	0.0/0.1	1.4/0.1	6.1/12.1	19.5/29.5
Social functioning	84.62 (17.24)	85.38 (19.17)	0.3/0.0	0.5/0.0	26.3/37.6	34.4/58.1
School functioning	77.72 (18.50)	77.80 (22.00)	0.3/0.0	1.7/0.3	8.5/17.9	15.5/34.5

### Discriminant validity

There were significant differences in reported HRQL between males and females (Pillai's trace = 0.012, p = 0.003) and across the chronic health conditions (Pillai's trace = 0.107, p = 0.000) for self-report. Age group was not significant (Pillai's trace = 0.006, p = 0.082). For proxy-report, no difference in reporting was detected between parents of males and females (Pillai's trace = 0.007, p = 0.188) or of different ages (Pillai's trace = 0.008, p = 0.094). Significant differences in reporting were confirmed across chronic health conditions (Pillai's trace = 0.219, p = 0.000). No interactions were found between any combination of these three factors for either self or proxy-report. Thus one-way ANOVAs were undertaken comparing the four chronic health conditions and healthy children for self and proxy-report (see table 3) and comparing males and females for self-report only.

**Table 3:** One-way ANOVA comparing chronically ill and healthy children: self and proxy-report

**Scale**	**Self-report**				**Proxy-report**			
	**N**	**Mean (sd)**	**df**	**F**	**N**	**Mean (sd)**	**df**	**F**
**Total Score**			4,1393	23.84			4,965	41.07
Asthma	99	75.31(16.90) ***			74	71.79(17.53)***		
Diabetes	124	82.46(12.76)			103	77.54(12.21) ***		
Cancer	66	75.68(15.40) ***			61	70.96(17.06) ***		
IBD	76	74.18(14.66) ***			67	72.65(17.62) ***		
Healthy	1033	83.89(11.84)			665	84.61(11.19)		
**Physical Health**			4,1392	41.60			4,964	46.87
Asthma	99	76.14(19.10)***			75	73.36(20.60) ***		
Diabetes	124	84.75(13.65)**			103	82.97(13.67) ***		
Cancer	66	78.10(17.64) ***			61	75.04(18.79) ***		
IBD	76	75.08(18.21) ***			67	71.54(21.98) ***		
Healthy	1032	88.51(11.62)			665	89.06(12.27)		
**Psychosocial Health**			4,1393	14.53			4,964	28.16
Asthma	99	74.9(17.48) ***			74	71.20(17.95) ***		
Diabetes	124	81.24(13.77)			103	74.62(13.27) ***		
Cancer	66	74.37(15.85) ***			61	68.83(17.92) ***		
IBD	76	73.64(14.35) ***			67	73.21(17.43) ***		
Healthy	1033	81.84(13.21)			664	82.21(12.67)		
**Emotional functioning**			4,1393	9.85			4,962	23.24
Asthma	99	70.66(20.06) ***			74	67.23(21.20) ***		
Diabetes	124	78.85(18.28)			102	66.01(17.80) ***		
Cancer	66	73.56(18.39) *			61	68.36(18.04) ***		
IBD	76	68.11(18.90) ***			67	67.26(21.41) ***		
Healthy	1033	78.49(17.94)			663	78.28(15.54)		
**Social functioning**			4,1393	5.89			4,964	14.09
Asthma	99	81.76(21.35) ***			74	76.96(21.69) ***		
Diabetes	124	89.15(13.91)			103	85.28(15.98)		
Cancer	66	81.27(18.36) **			61	73.28(22.93) ***		
IBD	76	83.82(16.61)			67	82.12(18.09) *		
Healthy	1033	87.65(16.46)			664	86.82(15.42)		
**School functioning**			4,1386	14.12			4,960	26.90
Asthma	99	72.37(19.62) ***			74	69.02(22.55) ***		
Diabetes	124	77.70(17.39)			103	72.62(17.64) ***		
Cancer	63	67.38(20.36) ***			58	63.45(21.71) ***		
IBD	73	69.52(17.28) ***			66	70.46(20.95) ***		
Healthy	1032	78.87(15.89)			664	81.52(16.09)		

* Denotes difference from healthy children at p < 0.05

* *Denotes difference from healthy children at p < 0.01

* **Denotes difference from healthy children at p < 0.001

Scores for children with a chronic health condition were lower than those for healthy children on all proxy-report scales, with most differences reaching significance (see table 3). For self-report, children with asthma, IBD and cancer survivors showed lower scores than healthy children on all scales, with most differences reaching significance (see table 3). In contrast, children with diabetes did not report lower HRQL than healthy children for all domains; for this group scores were higher than those of healthy children for emotional and social functioning, although this was not significant.

The only sub-scale on the self-report measure to show significant sex differences was Emotional Functioning, with females reporting lower HRQL than males (F (1,1396) = 29.66; p = 0.001). Although the mean score for female respondents at 74.39 (sd = 19.32) was lower than the male mean score of 79.71(sd = 17.04), these scores are still at the high end of the scale. The differences were however big enough to be reflected in both the composite psychosocial summary score (mean score: females = 79.65 (sd = 14.38), males = 81.39(sd = 13.68), F (1,1396) = 5.35; p = 0.021) and the total score (mean score: females = 81.32 (sd = 13.24), males = 83.21(sd = 12.89), F (1,1396) = 7.30; p = 0.007).

Table 4 shows the correlation between self and proxy-report. Moderate correlation was shown between the two forms on the same subscales, although correlations were higher for children with a chronic health condition than for healthy children.

**Table 4:** Correlation between self-report and proxy forms

Scale:	Total Sample	Healthy children	Children with chronic health condition
Total score	0.56*	0.32*	0.67*
Physical health	0.53*	0.20*	0.61*
Psychosocial health	0.51*	0.34*	0.63*
Emotional functioning	0.41*	0.27*	0.51*
Social functioning	0.50*	0.42*	0.60*
School functioning	0.49*	0.32*	0.56*

* Correlation is significant at P < 0. 001

## Discussion

The performance of the UK-English PedsQL™ (age range 8–18 years) was found to be similar to that reported for the original PedsQL™ [3]. We found excellent internal reliability of 0.90 for both self and proxy-report total scales, indicating the suitability of the total scale scores for individual patient analysis [9]. All subscale and summary scores exceeded 0.70, making them acceptable for group comparisons. This is comparable to the reliabilities reported for the original PedsQL™ of 0.88 and 0.90 for self and proxy-report total scales respectively, with all subscale and summary scores also exceeding 0.70[3].

As with the original PedsQL™, although no floor effects were found the existence of ceiling effects should be noted [3]. Thus whilst the full range of scoring options is used for the majority of subscales, responses tend to be skewed towards the top end of the scale for all subscales, for both self and proxy-report. However, it has been suggested ceiling and floor effects are to be expected in generic HRQL instruments, simply because they aim to be applicable to a wide range of populations [10]. It is possible that the health conditions of the children who took part in the study were well controlled, leading to better HRQL ratings. This issue should be explored further through the administration of PedsQL™ to children with a wider range of health issues including those experiencing acute health problems.

PedsQL™ performed as hypothesized using the known-groups method. There were differences in HRQL between healthy children and those with chronic health conditions for both proxy and self-report. However, children with diabetes scored significantly lower than healthy children on only one dimension – physical functioning. Indeed, they reported better HRQL than children with other chronic health problems and on social and emotional functioning rated their HRQL as better than healthy children, although this did not reach significance. The similarity in HRQL between children with diabetes and their healthy peers has been noted elsewhere [11]. Furthermore, a study into the impact of diabetes screening on adult HRQL reported similarities in HRQL of adults with and without diabetes – both before and after diagnosis [12]. This suggests that the findings of our study are neither atypical nor indicative of a problem with PedsQL™ measurement, but rather represent a meaningful difference in the HRQL of children with diabetes and those with other chronic health problems. Whether this is due to good disease management, the positive support of the diabetes care team or other factors remains unclear. What is apparent however, is that this issue merits further investigation.

Varni et al [3] did not report any differences in parental or child reporting of HRQL either by age or sex of the child. Whilst this study also found no differences in reporting for the proxy-report, a significant difference in male and female reporting of HRQL was found on the emotional functioning sub-scale of the self-report, with females reporting significantly lower levels of emotional functioning than males. The difference between males and females reflected in our data is consistent with much of the psychological literature concerning gender differences in emotional health [13,14]. In addition to suggesting that females are more likely to suffer more emotional health problems such as anxiety and depression than males, studies have also proposed that this gender difference is rooted in adolescence [15,16]. Furthermore, differences in male and female responses to illness have also been suggested, with females more likely to suffer depression following traumatic injury [17] and to display greater anxiety about chronic illness [18,19]. Thus the difference in emotional functioning we report here, would seem to reflect a genuine disparity between males and females and so offer further evidence for the validity of PedsQL™ as a sensitive measure of the emotional functioning of children and young people.

Moderate correlation was found between self and proxy-report. The pattern of parent-child correlation for the total sample is similar to that reported for the original PedsQL™, where better correlation was found for physical than for psychological and social functioning [3]. Yet, it should be noted that correlation is better between parents and children where the child has a chronic health condition. Indeed the most marked difference in correlation is on the physical health scale, suggesting that parents and children are more likely to share information about an issue if it is perceived as a problem (in this case physical health). Thus, whilstprevious research has found that parents and children agree more about physical problems, rather than internalising problems such as anxiety or sadness [20] this may depend in part on whether or not the child has a health problem. Furthermore, it is likely that proxy-reports reflect parental anxiety about their child; in this study parents of children with chronic health problems consistently underestimated their child's HRQL. The limited correlation observed between self and proxy-reports confirms the need to measure both child and parent perspectives when evaluating paediatric HRQL. Furthermore, in situations when the child is unable or unwilling to complete the self-report making it necessary to use a proxy-report to estimate HRQL, the knowledge that this estimate maybe inaccurate should be considered.

A potential limitation of this study is that retest reliability and responsiveness was not conducted. However, it has been argued that test-retest reliability may be less useful than internal consistency reliability in HRQL instrument development [21]. Internal consistency is suggested as a more valuable assessment of the reliability of a measure because of the likelihood of short-term fluctuations in health conditions such as those employed in this study, in which external factors such as disease and treatment variables are known to influence functioning.

## Conclusion

We have shown that the UK-English PedsQL™ is valid and reliable, replicating some of the previous findings for the generic PedsQL™ [3] for the first time with a UK population. The UK-English measure will be a valuable tool for assessing the HRQL of school-aged children in the UK, providing a useful outcome measure in both a research and clinical setting.

## Authors' contributions

PU made substantial contributions the acquisition of data, analysis and interpretation of data and the drafting of the article. CE made substantial contributions to the design of the study, the acquisition of data and interpretation of the data, drafting and revising the article. IC, MJ, AM, IR & JW all made substantial contributions to conception and design of the study and have been involved in revising the paper for important intellectual content. All authors have given final approval of the version to be published.

## Competing interests

The author(s) declare that they have no competing interests.
